# Gender-specific discrepancy in subjective global assessment for mortality in hemodialysis patients

**DOI:** 10.1038/s41598-018-35967-3

**Published:** 2018-12-14

**Authors:** Ye Eun Ko, Taeyoung Yun, Hye Ah Lee, Seung-Jung Kim, Duk-Hee Kang, Kyu Bok Choi, Yon Su Kim, Yong-Lim Kim, Hyung Jung Oh, Dong-Ryeol Ryu

**Affiliations:** 1 Ewha Womans University, College of Medicine, Seoul, Korea; 2 Clinical Trial Center, Ewha Womans University Mokdong Hospital, Seoul, Korea; 3 Department of Internal Medicine, College of Medicine, Ewha Womans University, Seoul, Korea; 4 Department of Internal Medicine, Seoul National University of Medicine, Seoul, Korea; 5 Department of Internal Medicine, Kyungpook National University School of Medicine, Daegu, Korea; 6 Ewha Institute of Convergence Medicine, Ewha Womans University Mokdong Hospital, Seoul, Korea; 7 Research Institute for Human Health Information, Ewha Womans University Mokdong Hospital, Seoul, Korea; 8 Tissue Injury Defense Research Center, Ewha Womans University, Seoul, Korea

## Abstract

Although subjective global assessment (SGA) is a widely used representative tool for nutritional investigations even among dialysis patients, no studies have examined gender-specific differences in the ability of SGA to predict mortality in hemodialysis (HD) patients. A total of 2,798 dialysis patients were enrolled from clinical research center for end-stage renal disease (CRC for ESRD) between 2009 and 2015. The cohort was divided into two groups based on nutritional status as evaluated by SGA: ‘good nutrition’ and ‘mild to severe malnutrition’. Multivariate Cox proportional regression analyses were performed to investigate gender-specific differences in SGA for mortality among incident and prevalent HD patients. ‘Mild to severe malnutrition’ was significantly correlated with increased mortality compared with ‘good nutrition’ for all HD, incident and prevalent HD patients. Compared with ‘good nutrition’, ‘mild to severe malnutrition’ was also more significantly associated with increased mortality in male patients in the incident and prevalent HD groups. However, no significant associations between nutritional status evaluated by SGA and mortality were observed for female patients. SGA of HD patients can be useful for predicting mortality, especially in male HD patients. However, SGA alone might not reflect adverse outcomes in female patients.

## Introduction

Malnutrition refers to an abnormal status originating from an inadequate diet and is well known to aggravate various clinical outcomes^1–4^. Moreover, it is rather common and has a higher prevalence in chronic dialysis patients than in the healthy population^5–9^. The Subjective Global Assessment (SGA) is a widely used representative tool for nutritional investigation; it is not only available for the nutritional assessment of dialysis patients but is also very practical and convenient for evaluating malnutrition in patients with end-stage renal disease (ESRD)^10,11^. However, the relationship between nutritional status evaluated by SGA and all-cause mortality has not been consistent across studies^12–14^.

Women in the general population have a longer life expectancy than men^15^. However, the survival rate for women in chronic dialysis treatment is similar to that of men undergoing dialysis^16–18^. Although the reasons for this observation have not been fully investigated, men have a somewhat higher estimated glomerular filtration rate (eGFR) at the start of dialysis than women^19–21^. Such gender differences at the start of dialysis may lead to the similar survival rates in dialysis patients^22^. Moreover, among patients undergoing hemodialysis (HD), men and women may have different multifactorial traits. However, there has been no study, to the best of our knowledge, on predicting mortality differences between male and female HD patients according to SGA. Thus, the aim of this study was to investigate gender-specific differences in SGA regarding all-cause mortality in an ESRD cohort in a Korean clinical research center.

## Results

### Baseline characteristics

Among the 2,798 patients, the mean age was 58.2 ± 13.8 years, 1,649 (58.9%) patients were male, and the mean body mass index (BMI) was 22.7 ± 3.5 kg/m^2^. There were 2,012 (71.9%) patients categorized as having a ‘good nutrition’ status as assessed by SGA at baseline. Moreover, 1,522 (54.4%) patients had diabetes mellitus (DM), 441 (15.8%) patients were suffering from coronary arterial disease (CAD), and 206 (7.4%) patients were diagnosed with peripheral arterial disease (PAD). Additionally, patients were coping with other comorbid diseases, as presented in Table 1. Regarding laboratory data, the mean hemoglobin, albumin, blood urea nitrogen (BUN), creatinine, total cholesterol, triglyceride, and high-sensitivity C-reactive peptide (hs-CRP) concentrations were 9.7 g/dL, 3.6 g/dL, 73.4 mg/dL, 9.1 mg/dL, 154.0 mg/dL, 122.6 mg/dL, and 3.5 mg/L, respectively.

**Table 1:** Baseline characteristics at enrollment according to nutritional status evaluated by SGA.

	Total (n = 2,798, 100%)	Good nutrition (n = 2,012, 71.9%)	Mild to severe malnutrition (n = 786, 28.1%)	P value
Age (years)	58.2 ± 13.8	57.3 ± 13.6	60.6 ± 13.9	<0.001
Gender (male, %)	1649 (58.9)	1220 (60.6)	429 (54.6)	0.003
Body mass index (kg/m^2^)	22.7 ± 3.5	22.7 ± 3.3	22.6 ± 3.8	0.20
Comorbidities				
Diabetes mellitus (%)	1522 (54.4)	1060 (52.7)	462 (58.8)	0.004
Coronary artery disease (%)	441 (15.8)	312 (15.5)	129 (16.4)	0.56
Peripheral vascular disease (%)	206 (7.4)	136 (6.8)	70 (8.9)	0.051
Congestive heart failure (%)	321 (11.5)	216 (10.7)	105 (13.4)	0.050
Cerebrovascular accident (%)	100 (3.6)	66 (3.3)	34 (4.3)	0.18
Chronic lung disease (%)	208 (7.4)	139 (6.9)	69 (8.8)	0.09
Liver disease (moderate to severe, %)	108 (3.9)	66 (3.3)	42 (5.3)	0.01
Laboratory				
Hemoglobin (g/dL)	9.7 ± 3.4	9.9 ± 3.8	9.3 ± 1.7	<0.001
Albumin (g/dL)	3.6 ± 0.6	3.7 ± 0.6	3.4 ± 0.6	<0.001
Blood urea nitrogen (mg/dL)	73.4 ± 32.7	72.1 ± 30.2	76.8 ± 38.3	0.001
Creatinine (mg/dL)	9.1 ± 3.8	9.4±3.6	8.4 ± 4.2	<0.001
Total cholesterol (mg/dL)	154.0 ±42.9	153.8 ± 40.0	154.6 ± 49.9	0.68
Triglyceride (mg/dL)	122.6 ± 79.1	121.6 ± 78.0	125.5 ± 82.0	0.30
hs-CRP (mg/L)	3.5 ± 14.5	2.7 ± 11.7	5.5 ± 19.9	<0.001

Data are presented as n(%) or the mean ± SD.

Abbreviations; SGA, subjective global assessment; hs-CRP, high-sensitivity C-reactive protein.

For the evaluation of SGA, a trained physician assessed a subset of 10 to 15 patients to determine interassessor SGA agreement. There was no significant interassessor discrepancy (data not shown).

When we stratified these patients according to nutritional status evaluated by SGA, the ‘mild to severe malnutrition’ group was significantly older and had more females, lower hemoglobin, serum albumin and creatinine levels, but higher BUN and hs-CRP levels than the ‘good nutrition’ group. Additionally, the ‘mild to severe malnutrition’ group had significantly more DM and moderate-to-severe liver disease (Table 1).

Table 2 presents the baseline characteristics at enrollment according to dialysis vintage. There was a higher percentage of male patients, ‘mild to severe malnutrition’ patients, DM, and PAD in the incident HD group than in the prevalent HD group, whereas there was a lower presence of CAD in the incident HD group. Moreover, BUN, total cholesterol, and hs-CRP values were significantly higher in the incident HD group than in the prevalent HD group, whereas hemoglobin, serum albumin, and creatinine levels were significantly lower in the incident HD patients than the prevalent HD patients (Table 2).

**Table 2:** Baseline characteristics at enrollment according to dialysis vintage.

	Total (n = 2,798, 100%)	Incident (n = 1,481, 52.9%)	Prevalent (n = 1,317, 47.1%)	P value
Age (years)	58.2 ± 13.8	58.2 ± 14.3	58.3 ± 13.2	0.74
Gender (male, %)	1,649 (58.9)	921 (62.2)	728 (55.3)	<0.001
Nutritional status				<0.001
Good nutrition (%)	2,012 (71.9)	916 (61.9)	1,096 (83.2)	
Mild to severe nutrition (%)	786 (28.1)	565 (38.1)	221 (16.8)	
Body mass index (kg/m^2^)	22.7 ± 3.5	23.1 ± 3.6	22.2 ± 3.3	0.10
Dialysis duration (months)	—	—	51.9 ± 48.0	—
Comorbidities				
Diabetes mellitus (%)	1,522 (54.4)	861 (58.1)	661 (50.2)	<0.001
Coronary artery disease (%)	441 (15.8)	213 (14.4)	228 (17.3)	0.03
Peripheral vascular disease (%)	206 (7.4)	126 (8.5)	80 (6.1)	0.01
Congestive heart failure (%)	321 (11.5)	177 (12.0)	144 (10.9)	0.40
Cerebrovascular accident (%)	100 (3.6)	42 (2.9)	58 (4.4)	0.07
Chronic lung disease (%)	208 (7.4)	113 (7.6)	95 (7.2)	0.68
Liver disease (moderate to severe, %)	108 (3.9)	49 (3.3)	59 (4.5)	0.11
Laboratory				
Hemoglobin (g/dL)	9.7 ± 3.4	8.8 ± 1.6	10.8 ± 4.3	<0.001
Albumin (g/dL)	3.6 ± 0.6	3.3 ± 0.6	3.9 ± 0.4	<0.001
Blood urea nitrogen (mg/dL)	73.4 ± 32.7	82.3 ± 38.9	63.3 ± 19.6	<0.001
Creatinine (mg/dL)	9.1 ± 3.8	8.6 ± 4.2	9.7 ± 3.1	<0.001
Total cholesterol (mg/dL)	154.0 ± 42.9	155.3 ± 49.1	152.7 ± 35.4	0.13
Triglyceride (mg/dL)	122.6 ± 79.1	124.5 ± 76.9	120.9 ± 81.1	0.26
hs-CRP (mg/L)	3.5 ± 14.5	4.9 ± 17.9	1.8 ± 9.0	<0.001

Data are presented as n(%) or the mean ± SD.

Abbreviations; hs-CRP, high-sensitivity C-reactive protein.

### The predictability of SGA for mortality according to dialysis vintage

**Figure 1:**
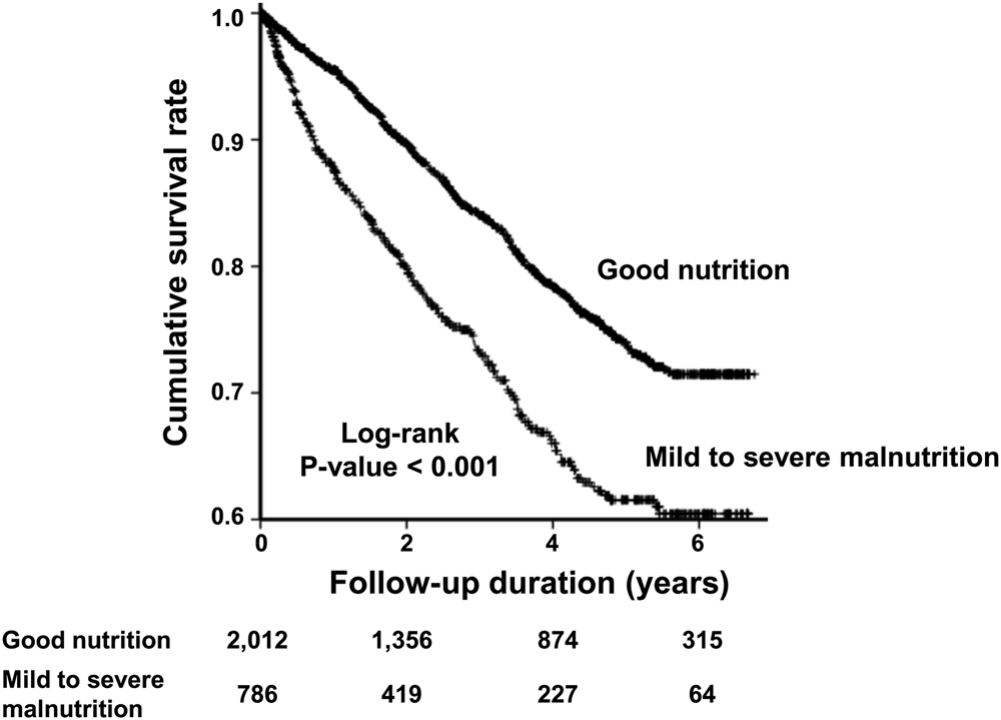
Kaplan-Meier curve for death in all hemodialysis patients. The Kaplan-Meier survival curve shows that the cumulative survival rate in G1 was significantly higher than that in G2 among all patients (*P* < 0.001).

**Figure 2:**
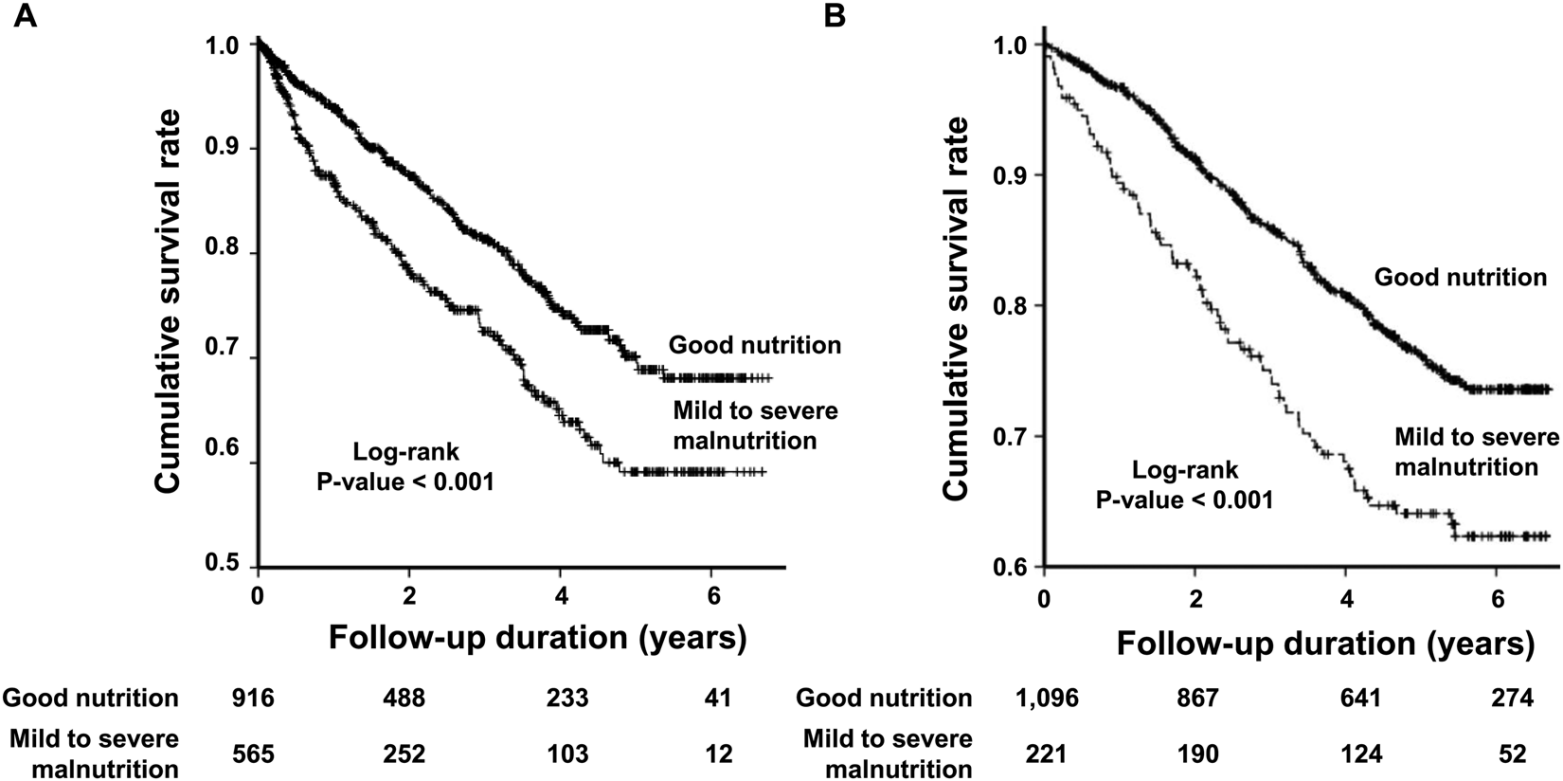
Kaplan-Meier curve for death in incident and prevalent hemodialysis patients. The Kaplan-Meier survival curve revealed that the cumulative survival rate in G1 was significantly higher than that in G2 among incident (**A**) and prevalent (**B**) hemodialysis patients (*P* < 0.001).

During the median 3.1 years of follow-up, 590 (21.1%) patients died. The Kaplan-Meier survival curve showed that the cumulative survival rate in the ‘good nutrition’ group was significantly higher than that in the ‘mild to severe malnutrition’ group among all the HD patients (*P* < 0.001) (Fig. 1) as well as among the incident (*P* < 0.001) (Fig. 2A) and prevalent (*P* < 0.001) HD groups (Fig. 2B).

As seen in Table 3, univariate Cox regression analysis showed that the hazard ratio (HR) for mortality in the ‘mild to severe malnutrition’ group was 1.73 (95% CI; 1.46–2.05, *P* < 0.001) compared with the ‘good nutrition’ group among all HD patients (Table 3A). Moreover, in the incident HD patients, the HR for mortality in the ‘mild to severe malnutrition’ group was 1.57 (95% CI; 1.25–1.98, *P* < 0.001 in Table 3B), and the HR in the prevalent HD patients was 1.70 (95% CI; 1.31–2.21, *P* < 0.001 in Table 3C). Furthermore, multivariate Cox proportional regression analyses revealed that the ‘mild to severe malnutrition’ group remained significantly associated with increased mortality even after adjusting for age, gender, the presence of DM, moderate-to-severe liver disease, and hemoglobin, serum albumin, BUN, and hs-CRP levels among all HD patients, incident patients, and prevalent patients when compared with the ‘good nutrition’ group [in all HD patients; hazard ratio (HR) = 1.34, 95% confidence interval (CI) = 1.13–1.60, *P* = 0.001, Table 3A, in the incident patients; HR = 1.28, 95% CI = 1.01–1.62, *P* = 0.04, Table 3B, and in the prevalent patients; HR = 1.37, 95% CI = 1.05–1.80, *P* = 0.02, Table 3C].

**Table 3:** Cox regression analysis of nutritional status and mortality among all HD patients.

	Univariate		Multivariate	
	HR (95% CI)	P-value	HR (95% CI)	P-value
**(A)**				
Nutritional status group				
Good nutrition	Reference	—	Reference	—
Mild to severe malnutrition	1.73 (1.46–2.05)	<0.001	1.34 (1.13–1.60)	0.001
Age (per 1 year increase)	1.06 (1.05–1.07)	<0.001	1.06 (1.05–1.06)	<0.001
Female (versus male)	0.71 (0.60–0.84)	<0.001	0.71 (0.60–0.84)	<0.001
DM (versus without DM)	1.75 (1.48–2.08)	<0.001	1.39 (1.17–1.65)	<0.001
MSLD (versus without MSLD)	1.76 (1.26–2.45)	0.001	1.62 (1.15–2.27)	0.005
Hemoglobin (per 1 g/dL increase)	0.97 (0.93–1.01)	0.19	1.00 (0.97–1.04)	0.80
Albumin ≥ 3.7 g/dL (vs.<3.7 g/dL)	0.54 (0.46–0.63)	<0.001	0.69 (0.58–0.82)	<0.001
Blood urea nitrogen (per 1 mg/dL increase)	1.00 (0.99–1.00)	0.03	1.00 (1.00–1.00)	0.15
hs-CRP ≥ 1.0 mg/L (vs.<1.0 mg/L)	1.93 (1.63–2.28)	<0.001	1.36 (1.14–1.63)	0.001
**(B) Cox regression analysis of nutritional status and mortality among incident HD patients**				
Nutritional status group				
Good nutrition	Reference	—	Reference	—
Mild to severe malnutrition	1.57 (1.25–1.98)	<0.001	1.28 (1.01–1.62)	0.04
Age (per 1 year increase)	1.06 (1.05–1.07)	<0.001	1.06 (1.05–1.07)	<0.001
Female (versus male)	0.85 (0.66–1.08)	0.17	0.83 (0.65–1.06)	0.14
DM (versus without DM)	1.63 (1.27–2.10)	<0.001	1.35 (1.04–1.74)	0.02
MSLD (versus without MSLD)	2.13 (1.32–3.44)	0.002	1.79 (1.10–2.90)	0.02
Hemoglobin (per 1 g/dL increase)	0.99 (0.93–1.07)	0.83	0.94 (0.87–1.02)	0.14
Albumin ≥ 3.7 g/dL (vs.<3.7 g/dL)	0.64 (0.49–0.85)	0.002	0.79 (0.59–1.05)	0.11
Blood urea nitrogen (per 1 mg/dL increase)	1.00 (0.99–1.00)	0.051	1.00 (1.00–1.00)	0.47
hs-CRP ≥ 1.0 mg/L (vs.<1.0 mg/L)	1.87 (1.48–2.35)	<0.001	1.34 (1.05–1.70)	0.02
**(C) Cox regression analysis of nutritional status and mortality among prevalent HD patients**				
Nutritional status group				
Good nutrition	Reference	—	Reference	—
Mild to severe malnutrition	1.70 (1.31–2.21)	<0.001	1.37 (1.05–1.80)	0.02
Age (per 1 year increase)	1.06 (1.05–1.07)	<0.001	1.06 (1.04–1.07)	<0.001
Female (versus male)	0.64 (0.50–0.81)	<0.001	0.61 (0.48–0.78)	<0.001
DM (versus without DM)	1.78 (1.40–2.25)	<0.001	1.44 (1.14–1.82)	0.003
MSLD (versus without MSLD)	1.57 (0.99–2.50)	0.06	1.42 (0.88–2.29)	0.16
Hemoglobin (per 1 g/dL increase)	1.00 (0.97–1.03)	0.93	1.02 (0.99–1.04)	0.16
Albumin ≥ 3.7 g/dL (vs.<3.7 g/dL)	0.48 (0.37–0.61)	<0.001	0.66 (0.51–0.85)	0.001
Blood urea nitrogen (per 1 mg/dL increase)	0.99 (0.98–1.00)	<0.001	0.99 (0.99–1.00)	0.02
hs-CRP ≥ 1.0 mg/L (vs.<1.0 mg/L)	1.81 (1.40–2.34)	<0.001	1.37 (1.06–1.78)	0.02

Abbreviations; HD, hemodialysis; HR, hazard ratio; CI, confidence interval.

Adjusted for age, gender, the presence of diabetes mellitus, liver disease, hemoglobin, albumin, blood urea nitrogen, and high-sensitivity C-reactive protein.

Abbreviations; HD, hemodialysis; HR, hazard ratio; CI, confidence interval.

Adjusted for age, gender, the presence of diabetes mellitus, liver disease, hemoglobin, albumin, blood urea nitrogen, and high-sensitivity C-reactive protein.

Abbreviations; HD, hemodialysis; HR, hazard ratio; CI, confidence interval.

Adjusted for age, gender, the presence of diabetes mellitus, liver disease, hemoglobin, albumin, blood urea nitrogen, and high-sensitivity C-reactive protein.

### Gender-specific discrepancy in SGA for mortality

We performed multivariate Cox proportional regression analyses to reveal gender-specific discrepancies in SGA for mortality among the incident and prevalent HD patients. Univariate analysis showed that compared with the ‘good nutrition’ group, the ‘mild to severe malnutrition’ group was significantly correlated with increased mortality in males in both the incident and prevalent HD patients (in incident patients; HR = 2.00, 95% CI = 1.50–2.67, *P* < 0.001, and in prevalent patients; HR = 2.03, 95% CI = 1.45–2.84, *P* < 0.001), and the ‘mild to severe malnutrition group’ remained significantly associated with increased mortality in males in the incident and prevalent HD patients even after adjusting for age, the presence of DM, moderate-to-severe liver disease, and hemoglobin, serum albumin, BUN, and hs-CRP levels (in incident patients; HR = 1.56, 95% CI = 1.16–2.10, *P* = 0.003 and in prevalent patients; HR = 1.67, 95% CI = 1.18–2.36, *P* = 0.004). However, in females, no significant associations between nutritional status evaluated by SGA and mortality were observed (Table 4).

**Table 4:** Cox regression analysis of nutritional status and mortality according to gender among incident HD patients.

	Male				Female			
	Univariate		Multivariate		Univariate		Multivariate	
	HR (95% CI)	P-value	HR (95% CI)	P-value	HR (95% CI)	P-value	HR (95% CI)	P-value
**(A)**								
Nutritional status group								
Good nutrition	Reference	—	Reference	—	Reference	—	Reference	—
Mild to severe malnutrition	2.00 (1.50–2.67)	<0.001	1.56 (1.16–2.10)	0.003	1.11 (0.75–1.64)	0.60	0.93 (0.62–1.39)	0.73
Age (per 1 year increase)	1.05 (1.04–1.07)	<0.001	1.05 (1.03–1.06)	<0.001	1.07 (1.05–1.09)	<0.001	1.07 (1.05–1.09)	<0.001
DM (versus without DM)	1.90 (1.37–2.64)	<0.001	1.50 (1.07–2.10)	0.02	1.28 (0.86–1.91)	0.23	1.12 (0.74–1.68)	0.60
MSLD (versus without MSLD)	1.88 (1.09–3.24)	0.02	1.68 (0.97–2.92)	0.06	2.93 (1.08–7.99)	0.04	2.07 (0.74–5.79)	0.17
Hemoglobin (per 1 g/dL increase)	1.00 (0.92–1.09)	0.97	0.97 (0.88–1.07)	0.59	0.98 (0.87–1.11)	0.77	0.89 (0.78–1.02)	0.10
Albumin ≥ 3.7 g/dL (vs.<3.7 g/dL)	0.66 (0.47–0.93)	0.02	0.85 (0.60–1.22)	0.38	1.63 (1.02–2.61)	0.04	0.68 (0.42–1.11)	0.13
Blood urea nitrogen (per 1 mg/dL increase)	1.00 (0.99–1.00)	0.06	1.00 (0.99–1.00)	0.39	1.00 (0.99–1.00)	0.29	1.00 (1.00–1.01)	0.74
hs-CRP ≥ 1.0 mg/L (vs.<1.0 mg/L)	1.86 (1.39–2.47)	<0.001	1.37 (1.01–1.86)	0.04	1.84 (1.24–2.72)	0.002	1.36 (0.91–2.05)	0.14
**(B) Cox regression analysis of nutritional status and mortality according to gender among prevalent HD patients**								
Nutritional status group								
Good nutrition	Reference	—	Reference	—	Reference	—	Reference	—
Mild to severe malnutrition	2.03 (1.45–2.84)	<0.001	1.67 (1.18–2.36)	0.004	1.42 (0.93–2.16)	0.11	1.05 (0.68–1.62)	0.82
Age (per 1 year increase)	1.07 (1.05–1.08)	<0.001	1.06 (1.05–1.08)	<0.001	1.05 (1.04–1.07)	<0.001	1.05 (1.03–1.06)	<0.001
DM (versus without DM)	2.42 (1.77–3.31)	<0.001	1.41 (1.04–1.90)	0.03	2.46 (1.64–3.68)	<0.001	1.46 (0.99–2.15)	0.05
MSLD (versus without MSLD)	1.04 (0.63–1.70)	0.89	1.50 (0.91–2.49)	0.11	0.43 (0.06–3.12)	0.40	0.94 (0.13–6.79)	0.95
Hemoglobin (per 1 g/dL increase)	0.92 (0.83–1.02)	0.09	0.98 (0.89–1.08)	0.70	1.01 (0.99–1.04)	0.36	1.02 (1.00–1.04)	0.13
Albumin ≥ 3.7 g/dL (vs.<3.7 g/dL)	0.58 (0.42–0.81)	0.001	0.71 (0.51–0.99)	0.04	0.49 (0.33–0.72)	<0.001	0.59 (0.40–0.88)	0.01
Blood urea nitrogen (per 1 mg/dL increase)	0.99 (0.98–1.00)	0.003	1.00 (0.99–1.00)	0.36	0.98 (0.97–0.99)	<0.001	0.99 (0.98–1.00)	0.01
hs-CRP ≥ 1.0 mg/L (vs.<1.0 mg/L)	1.93 (1.40–2.65)	<0.001	1.47 (1.06–2.04)	0.02	1.41 (0.91–2.18)	0.12	1.19 (0.76–1.85)	0.45

Abbreviations; HD, hemodialysis; HR, hazard ratio; CI, confidence interval.

Adjusted for age, gender, the presence of diabetes mellitus, liver disease, hemoglobin, albumin, blood urea nitrogen, and high-sensitivity C-reactive protein.

Abbreviations; HD, hemodialysis; HR, hazard ratio; CI, confidence interval.

Adjusted for age, gender, the presence of diabetes mellitus, liver disease, hemoglobin, albumin, blood urea nitrogen, and high-sensitivity C-reactive protein.

## Discussion

In this study, SGA was significantly correlated with increased all-cause mortality in Korean HD patients, especially male patients. However, in female patients, we did not find significant associations between the nutritional status evaluated by SGA and mortality.

SGA was originally developed to identify poor nutritional status in subjects undergoing gastrointestinal surgery^23,24^ but has been adapted for use in patients with chronic and end-stage renal failure and has been used to quantify the prevalence of malnutrition in dialysis patients^10^. Although an abnormal SGA score predicts increased mortality in peritoneal dialysis (PD) patients^25–27^, there have been concerns regarding the use of SGA in practice. For example, Jones *et al*.^12^ and Cooper *et al*.^13^ demonstrated that SGA may not reliably identify HD patients with abnormal nutrition. Moreover, Kwon *et al*.^28^ reported that the impact of SGA on all-cause mortality was not significant in multivariate analysis. However, in this study, the nutritional status evaluated by SGA was significantly associated with increases in mortality in all HD patients, i.e., incident and prevalent HD patients.

In contrast, we found a gender-specific discrepancy in SGA for mortality in the current study. However, the reasons for this discrepancy are unclear. Most prior studies on SGA did not show gender-specific discrepancies in SGA for mortality. However, Liu *et al*.^29^ recently reported gender-specific associations of skeletal muscle mass and arterial stiffness among peritoneal patients. Although the current study was conducted with HD patients and we could not investigate their skeletal muscle mass, gender-specific characteristics may be present in skeletal muscle mass. This could be a factor influencing the gender-specific discrepancy in SGA for mortality. We surmise that there may be less skeletal muscle mass in female patients than in male patients. Female patients may be involved in lower levels of physical activity, and SGA alone may not fully reflect nutritional status in females compared with males.

Even though women in the general population have a longer life expectancy than men^15^, women undergoing chronic dialysis have as poor survival as men undergoing dialysis^16–18^. However, this observation may be biased, as the reasons for this observation have not been fully investigated. Interestingly, men have a somewhat higher eGFR at the start of dialysis than women do^19–21^; this may be attributed to gender differences in physician’s clinical judgement, although this has not been demonstrated^22^. We investigated the causes of death during the follow-up. Among a total of 590 mortalities, 289 patients were incident HD patients, and 301 patients were prevalent HD patients. As seen in Supplementary Tables 1 and 2, no significant differences in the cause of death between incident and prevalent HD patients were observed except for liver-associated death (P = 0.04). Additionally, no significant differences in the causes of death between the high and low SGA score groups in the incident and prevalent HD groups were observed. However, when we investigated the causes of death in male and female patients, we found that female HD patients (9.4%) had a vascular origin of death (e.g., cerebrovascular and peripheral vascular events) significantly more often than male HD patients (5.0%); this could indicate that death was less-correlated with nutritional status in female patients than in male patients (data not shown). Thus, the gender-specific discrepancy in SGA for mortality should be interpreted with caution; larger prospective cohort studies are necessary.

Huang *et al*.^30^ reported that gender could modify the influence of BMI on mortality in advanced chronic kidney disease (CKD) patients. This suggests that different nutritional indices based on gender may be needed to evaluate nutritional status in advanced CKD patients.

There are several limitations in this study. First, this study was limited to Korean HD patients. Thus, one should be cautious when interpreting the implications of this study and when generalizing our results to other ethnic groups. Jin and Han^31^ demonstrated the characteristics of Korean dialysis patients in the Korean Society of Nephrology (KSN). They examined ESRD patients (n = 70,211) and HD patients (n = 48,531, 69.1%). The prevalence of ESRD was 1,353.3 per million persons. The number of new ESRD patients in 2012 was 11,742 (HD, 8,811), the incidence rate was 221.1 per million persons. Moreover, the 5-year survival rates of male and female dialysis patients were 70.6% and 73.5%, respectively. Compared with Jin’s report, the mean age, proportion of males, and presence of DM were similar to those of the current manuscript. Moreover, our reported mortality rate was 21.1% during the median 3.1-year follow-up, which was similar to Jin’s 5-year mortality rates. Thus, we surmise that the patients in our study showed similar general characteristics to Korean HD patients. Second, SGA was assessed and laboratory data were measured only once at the time of enrollment. Therefore, it was difficult to statistically evaluate the association between SGA and mortality. Third, we arbitrarily combined ‘mild to moderate malnutrition’ and ‘severe malnutrition’ into one group, ‘mild to severe malnutrition’, because only a few patients (11 patients) were originally categorized in the ‘severe malnutrition’ group. Moreover, the patients were not evenly distributed. Fourth, SGA was evaluated when the patients were considered clinically stable and in a euvolemic state. Patients were assessed and scores were reported by an experienced dietician in each center, and a trained physician assessed subsets of 10 to 15 patients in each center to evaluate the degree of interassessor agreement. However, uremia could cause a lower SGA score in incident HD patients than in prevalent HD patients. Sixth, the statistical power was weak despite the significance of the data. Therefore, a larger data set will be needed to overcome this limitation. However, despite these limitations, the present study was the first to investigate a gender-specific discrepancy in SGA for mortality in a large cohort.

In conclusion, SGA can be useful for predicting mortality in HD patients, especially in male HD patients, whereas SGA alone may not reflect adverse outcomes in female patients. Further evaluation will be needed to explain this discrepancy.

## Subjects and Methods

### Patients

**Figure 3:**
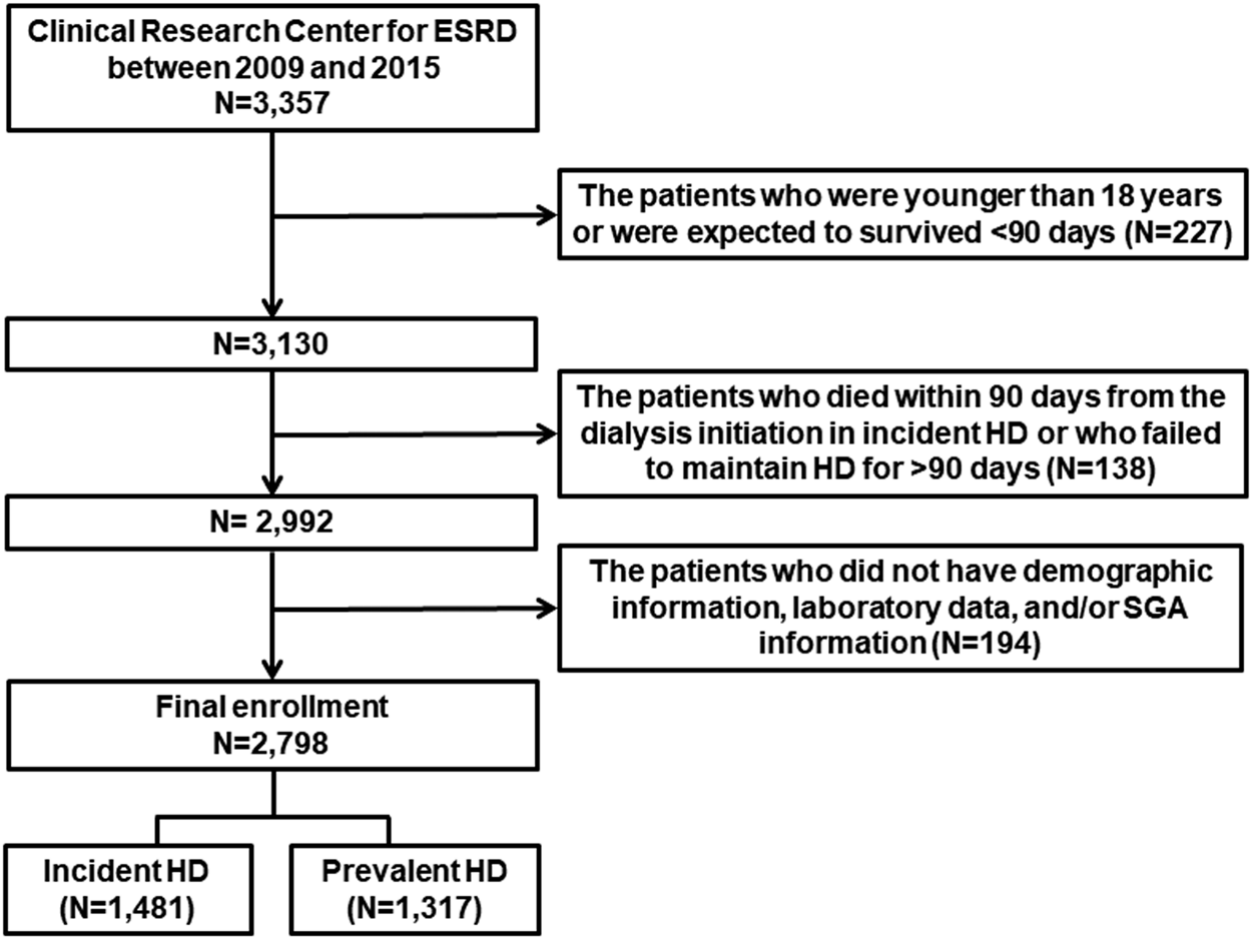
Flow diagram for the patients enrolled in this study.

All ESRD patients undergoing HD between May 2009 and December 2015 at one of the 36 centers of the Clinical Research Center for ESRD in Korea were recruited for this prospective observational multicenter study. This study was part of a nationwide multicenter joint network prospective cohort study on ESRD patients in Korea that was designed to improve survival rates and quality of life and to establish effective treatment guidelines (clinicaltrial.gov NCT00931970). We excluded patients younger than 18 years or who were expected to survive <90 days. Patients who died within 90 days of the initiation of dialysis in incident HD or who failed to maintain HD for >90 days were excluded. Moreover, patients who did not have demographic information, laboratory data, and/or SGA information were excluded. A total of 2,798 HD patients from the initially screened 3,357 patients were divided into two groups according to dialysis vintage, i.e., incident HD patients (n = 1,481) and prevalent HD patients (n = 1,317), to explore the effectiveness of SGA in predicting mortality in the two groups (Fig. 3).

The study protocol was approved by the Institutional Review Board of each participating center, and all patients provided written informed consent to participate in the study.

### Data Collection

Demographics and clinical data, including age, gender, BMI, and comorbid diseases, were investigated at the time of enrollment. We determined the presence of CAD when the patient had a history of angina, myocardial infarction, coronary angioplasty, or coronary artery bypass grafts; cerebrovascular disease when they had undergone transient ischemic attack, stroke, or carotid endarterectomy; and PAD when there was a history of claudication, any peripheral revascularization procedure, ischemic limb loss, and/or ulceration. Chronic lung disease (CLD) included the conditions of chronic obstructive pulmonary disease (COPD), sleep-disordered breathing, and interstitial lung disease. Moderate-to-severe liver disease was defined as chronic hepatitis with elevated liver function test results, symptomatic chronic active hepatitis requiring medication, esophageal varices, ascites, liver cirrhosis, history of portocaval shunts, or previous surgical procedure for portal hypertension.

Laboratory data were collected from fasting blood samples before the start of HD in a midweek session: hemoglobin, serum albumin, BUN, creatinine, total cholesterol, triglycerides, and hs-CRP levels were measured. Body weight was measured and recorded before dialysis on the same day that laboratory data were retrieved.

The Institutional Review Board of the Ewha Womans University Mokdong Hospital (EUMC 2015–05–049) approved this study. Additionally, all methods were performed in accordance with the relevant guidelines and regulations.

### Subjective Global Assessment

SGA was conducted when the patients were considered clinically stable and in a euvolemic state. SGA was evaluated and reported by an experienced dietician at each center, and a trained physician assessed a subset of 10 to 15 patients at each center to evaluate the degree of interassessor agreement.

The nutritional status of patients was examined using the 7-point SGA scale, which contained medical history and physical examinations. The medical history consisted of four categories: weight loss, gastrointestinal symptoms, functional capacity, and comorbidities^10^. The physical examination included loss of subcutaneous fat, muscle wasting, and edema^6^. Each component was rated from 1 to 7, and the overall SGA score was determined. Based on the overall SGA score, patients were classified into three groups: A = SGA score 6–7 (well nourished), B = SGA score 3–5 (mildly to moderately malnourished), or C = SGA score 1–2 (severely malnourished). Because only 11 patients were categorized in the C (severely malnourished) group, they were combined with the B group. The two remaining groups were designated ‘Good nutrition’ (SGA A) or ‘Mild to Severe malnutrition’ (SGA B + SGA C) (Supplementary Table 3).

### Outcome Measures

All patients were followed up prospectively after all baseline assessments. All mortality events were retrieved from the database and carefully reviewed. The primary endpoint was all-cause mortality. Loss to follow-up, renal transplantation, transfer to PD, and recovery of renal function after the first three months of dialysis commencement were censored at the end of each dialysis treatment. When a patient died within three months after being transferred to PD, the death was regarded as a mortality event of HD.

### Statistical analysis

Statistical analyses were performed using SPSS for Windows, version 20 (SPSS Inc., Chicago, IL). Continuous variables were expressed as the means ± standard deviations and categorical variables as numbers (percentage). According to the nutritional status evaluated by SGA, the patients were divided into two groups: ‘good nutrition’ and ‘mild to severe nutrition’. We also divided the patients into incident HD patients and prevalent HD patients based on dialysis vintage. The baseline characteristics were compared between the two groups using Student’s t-test for continuous variables and the χ^2^ test for categorical variables. Cumulative survival curves were created by the Kaplan-Meier method, and the survival was compared by a log-rank test. We tried to determine the effect of nutritional status evaluated by SGA on mortality according to dialysis vintage and gender. Thus, multivariate proportional regression analyses were also conducted to assess the association between nutritional status evaluated by SGA and mortality in each group. We computed scaled Schoenfeld residuals, and age was available for the Cox proportional hazards model as a linear variable. However, serum albumin and hs-CRP levels were not available for proportional hazard models as linear variables. Thus, serum albumin level was categorized based on the median value (3.7 g/dL) and adjusted for multivariate Cox analysis. Additionally, hs-CRP levels were categorized based on a normal ranged value (1.0 mg/dL). Finally, the ‘mild to severe malnutrition’ group had a significant association with increased mortality compared with the ‘good nutrition’ group even after adjusting for age, gender, the presence of DM and moderate-to-severe liver disease, and hemoglobin, albumin ≥3.7 g/dL, BUN, hs-CRP ≥ 1.0 mg/dL levels among all HD patients, incident and prevalent. HRs and 95% CIs were used to provide the relative risk of death of the enrolled patients. A *P* value less than 0.05 was considered statistically significant.

## Electronic supplementary material

Supplementary Tables
