# Supplementary material for: Gender-specific discrepancy in subjective global assessment for mortality in hemodialysis patients
Source: Sci Rep. 2018 Dec 14;8:17846. doi: 10.1038/s41598-018-35967-3 (PMC6294808; doi:10.1038/s41598-018-35967-3)
Supplement: Supplementary file 1 — Supplementary Tables [file 41598_2018_35967_MOESM1_ESM.doc]

**Gender-specific discrepancy of subjective global assessment for mortality in hemodialysis patients**

Ye Eun Ko^1*^, Taeyoung Yun^1*^, Hye Ah Lee^2^, Seung-Jung Kim^3^, Duk-Hee Kang^3^, Kyu Bok Choi^3^, Yon Su Kim^4^, Yong-Lim Kim^5^, Hyung Jung Oh^6,7†^, and Dong-Ryeol Ryu^3,6,7†^

^1^Ewha Womans University, College of Medicine, Seoul, Korea

^2^Clinical Trial Center, Ewha Womans University Mokdong Hospital, Seoul, Korea

^3^Department of Internal Medicine, College of Medicine, Ewha Womans University, Seoul, Korea

^4^Department of Internal Medicine, Seoul National University of Medicine, Seoul, Korea

^5^Department of Internal Medicine, Kyungpook National University School of Medicine, Daegu, Korea

^6^Ewha Institute of Convergence Medicine and ^7^Research Institute for Human Health Information, Ewha Womans University Mokdong Hospital, Seoul, Korea

^7^Tissue Injury Defense Research Center, Ewha Womans University, Seoul, Korea

**Running Title:** Gender-specific discrepancy of SGA for mortality

**Corresponding Author:**

^†^Hyung Jung Oh, MD, PhD

Ewha Institute of Convergence Medicine, Ewha Womans University Mokdong Hospital, Seoul, Korea, Tel: +82 2 2650 2907

E-mail: ohjmd@naver.com

^†^Dong-Ryeol Ryu, MD, PhD

Department of Internal Medicine, School of Medicine, Ewha Womans University, Seoul, Korea

Tel: +82 2 2650 2507

Fax: +82 2 2650 2501

E-mail: drryu@ewha.ac.kr

**^*^ Ko YE and Yun TY contributed equally to this study.**

| **Supplementary Table 1. Cause of death in incident and prevalent HD patients during the follow-up duration** | | | | |
| --- | --- | --- | --- | --- |
| **Cause of death** | **Total**  **(n=590,100%)** | **Incident**  **(n=289, 49.0%)** | **Prevalent**  **(n=301, 51.0%)** | **P value** |
| **Cardiac origin** | 123 (20.8) | 52 (18.0) | 71 (23.6) | 0.09 |
| **Vascular** | 39 (6.6) | 18 (6.2) | 21 (7.0) | 0.72 |
| **Infection** | 107 (18.1) | 53 (18.3) | 54 (17.9) | 0.90 |
| **Liver disease** | 8 (1.4) | 1 (0.3) | 7 (2.3) | 0.04 |
| **Gastrointestinal** | 6 (1.0) | 2 (0.7) | 4 (1.3) | 0.44 |
| **Metabolic** | 9 (1.5) | 5 (1.7) | 4 (1.3) | 0.69 |
| **Other** | 70 (11.9) | 34 (11.8) | 36 (12.0) | 0.94 |

Abbreviations; HD, hemodialysis

Cardiac origin was defined as the death associated with heart, such as cardiovascular events, heart failure, etc.

Vascular origin was defined as the death related to cerebrovascular events, peripheral vascular events.

**Supplementary Table 2. Cause of death according to nutritional status in each incident and prevalent HD group**

|  | **Incident** | | | | **Prevalent** | | | |
| --- | --- | --- | --- | --- | --- | --- | --- | --- |
| **Cause of death** | **Total**  **(n=289, 100%)** | **Good nutrition**  **(n=159, 55.0%)** | **Mild to severe Malnutrition**  **(n=130, 45.0%)** | **P value** | **Total**  **(n=301, 100%)** | **Good nutrition**  **(n=226, 75.1%)** | **Mild to severe Malnutrition**  **(n=75, 24.9%)** | **P value** |
| **Cardiac** | 52 (18.0) | 32 (20.1) | 20 (15.4) | 0.30 | 71 (23.6) | 57 (25.2) | 14 (18.7) | 0.25 |
| **Vascular** | 18 (6.2) | 9 (5.7) | 9 (6.9) | 0.66 | 21 (7.0) | 17 (7.5) | 4 (5.3) | 0.52 |
| **Infection** | 53 (18.3) | 28 (17.6) | 25 (19.2) | 0.72 | 54 (17.9) | 37 (16.4) | 17 (22.7) | 0.22 |
| **Liver disease** | 1 (0.3) | 1 (0.6) | 0 (0.0) | 0.37 | 7 (2.3) | 4 (1.8) | 3 (4.0) | 0.27 |
| **Gastrointestinal** | 2 (0.7) | 2 (1.3) | 0 (0.0) | 0.20 | 4 (1.3) | 2 (0.9) | 2 (2.7) | 0.24 |
| **Metabolic** | 5 (1.7) | 3 (1.9) | 2 (1.5) | 0.82 | 4 (1.3) | 2 (0.9) | 2 (2.7) | 0.24 |
| **Other** | 34 (11.8) | 18 (11.3) | 16 (12.3) | 0.80 | 36 (12.0) | 23 (10.2) | 13 (17.3) | 0.10 |

Abbreviations; HD, hemodialysis

**Supplementary Table 3**. Subjective Global Assessment

| ***Subjective Global Assessment (SGA) rating form*** | | | | | | | Date of assessment | | | | | | | | YYYY MM DD | | |
| --- | --- | --- | --- | --- | --- | --- | --- | --- | --- | --- | --- | --- | --- | --- | --- | --- | --- |
|  | | | | | | |  | | | | | | | |  | | |
| A. Medical History | | | | | | | | | | | | | | | | | SGA rating(1~7) |
| **1. Weight/Weight Change** | | | | | | | | | | | | | | | | |  |
| Weight loss in past 6 mo: | | |  | |  | | --- | | | kg | | |  | | --- | | | | | % | |  | | |
|  | | |  | □ < 5% | | □ 5~10% | | | | | □ >10% | | | | |  |
| Wt change in past two weeks: | | |  | □  □  □ | | Increase (gain)  No change (stabilization)  Decrease (continued loss) | | | | | | | | | | |
| **2. Dietary Intake** | | |  |  | |  | | | | |  | | | | | |  |
| Overall change: | | |  | □  □ | | No change  Change (Increase or Decrease) | | | | | | | | | | |
| Duration: | | |  | | |  | | --- | | | --- | --- | | | Weeks | | | | |  | | | | | |
| Diet change: | | |  | □  □  □  □ | | Suboptimal solid diet (75, 50, 25% intake)  Full liquid diet  Hypocaloric liquids  Starvation | | | | | | | | | | |
| **3. Gastrointestinal symptoms (persisting daily for 2 weeks)** | | | | | | | | | | | | | | | | |  |
|  | | |  | □  □  □  □  □ | | None  Vomiting  Diarrhea  Anorexia  Dysphagia/Odynophagia | | | | | | | | | | |
| **4. Functional Impairment** | | |  |  | |  | | | | | | | | | | |  |
| Overall impairment: | | |  | □  □  □ | | None ( full capacity)  Mild  Severe | | | | | | | | | | |
| Duration: | | |  | | |  | | --- | | | --- | --- | | | Weeks | | | | | | | | | | |
| Type: | | |  | □  □ | | Ambulatory (Walking or Wheelchair)  Bedridden | | | | | | | | | | |
| **5. Disease state/ comorbidities as related to nutritional needs** | | | | | | | | | | | | | | | | |  |
| Primary diagnosis : | |  | | --- | | | | | Comorbidities: | | | | |  | | --- | | | | | | | | |
| Metabolic burden | | |  | □  □  □  □ | | No stress  Minimal  Moderate  Severe | | | |  | | |  | | | |
| **B. Physical Examination** (for each trait specify: 1=normal, 2=mild-moderate, 3=severe) | | | | | | | | | | | | | | | | | |
| Loss of subcutaneous fat: | | |  | |  | | --- | | | (below eye, triceps, chest, biceps) | | | | | | | | | | |  |
| Muscle wasting: | | |  | |  | | --- | | | (Temple, Clavicle, scapula, ribs, quadriceps, calf, knee, interosseous) | | | | | | | | | | |
| Ankle edema | | |  | |  | | --- | | |  | | | | | | | | | | |
| Sacral edema | | |  | |  | | --- | | |  | | | | | | | | | | |
| Ascites | | |  | |  | | --- | | | (Hemodialysis only related) | | | | | | | | | | |
| **C. Overall SGA rating**(7 point) | | | | | | | | | | | | | | | | |  |
| **D. SGA rating** | | | | | | | | | | | | | | | | | |
| □ A. Well-nourished (6, 7) | | □ B. Mild-moderated malnourished (3,4,5) | | | | | | | | | | | | | □ C. Severe malnourished (1,2) | | |
